# An experimental study of muscular injury repair in a mouse model of notexin-induced lesion with EPI® technique

**DOI:** 10.1186/s13102-015-0002-0

**Published:** 2015-04-17

**Authors:** Ferran Abat, Soraya-L Valles, Pablo-Eduardo Gelber, Fernando Polidori, Adrian Jorda, Sergio García-Herreros, Joan-Carles Monllau, Jose-Manuel Sanchez-Ibáñez

**Affiliations:** 1 Department of Sports Orthopedics, ReSport Clinic, Barcelona, Spain; 2 Department of Physiology, Faculty of Medicine, University of Valencia, Valencia, Spain; 3 Catalan Institut of Traumatology and Sports Medicine (ICATME), Hospital Universitari Dexeus, Universitat Autónoma de Barcelona, Barcelona, Spain; 4 Department of Orthopedic Surgery, Hospital de la Santa Creu i Sant Pau, University Autonoma of Barcelona, Barcelona, Spain; 5 Department of Sports Rehabilitation, Cerede Sports Medicine, Barcelona, Spain; 6 Universitat Autónoma de Barcelona, Barcelona, Spain; 7 Department of Orthopedic Surgery and Traumatology, Hospital del Mar, Universitat Autónoma de Barcelona, Barcelona, Spain

**Keywords:** EPI, Technique, Notexin-induced, Muscle, Injury

## Abstract

**Background:**

The mechanisms of muscle injury repair after EPI® technique, a treatment based on electrical stimulation, have not been described. This study determines whether EPI® therapy could improve muscle damage.

**Methods:**

Twenty-four rats were divided into a control group, Notexin group (7 and 14 days) and a Notexin + EPI group. To induce muscle injury, Notexin was injected in the quadriceps of the left extremity of rats. Pro-inflammatory interleukin 1-beta (IL-1beta) and tumoral necrosis factor-alpha (TNF-alpha) were determined by ELISA. The expression of receptor peroxisome gamma proliferator activator (PPAR-gamma), vascular endothelial growth factor (VEGF) and vascular endothelial growth factor receptor-1 (VEGF-R1) were determined by western-blot.

**Results:**

The plasma levels of TNF-alpha and IL-1beta in Notexin-injured rats showed a significant increase compared with the control group. EPI® produced a return of TNF-alpha and IL-1beta values to control levels. PPAR-gamma expression diminished injured quadriceps muscle in rats. EPI® increased PPAR-gamma, VEGF and VEGF-R1 expressions. EPI® decreased plasma levels of pro-inflammatory TNF-alpha and IL-1beta and increased anti-inflammatory PPAR-gamma and proangiogenic factors as well as VEGF and VEGF-R1 expressions.

**Conclusion:**

The EPI® technique may affect inflammatory mediators in damaged muscle tissue and influences the new vascularization of the injured area. These results suggest that EPI® might represent a useful new therapy for the treatment of muscle injuries. Although our study in rats may represent a valid approach to evaluate EPI® treatment, studies designed to determine how the EPI® treatment may affect recovery of injury in humans are needed.

## Background

Soft tissue injuries are recurrent in sports and have an incidence rate of some 30% [1]. An overly conservative therapeutic approach conflicts with patients’ economics interests and the ability to practice their chosen sport. Some authors have proposed qualitative and histopathological classifications of muscle injuries directly related to the appearance of the lesion and its evolution [2].

The inflammatory process is one of the most important parts of the immune system’s response to injury. It is due to the fact that the biochemical mechanism and the signal cascade are consistent and durable, independent of the underlying cause of the wound [3]. Non-muscle cells such as leukocytes, phagocytes, macrophages, cytokines or growth factors play an important role in the inflammatory process in terms of recovery and regeneration following injury to the muscle as well as in the secondary damage that occurs during the inflammatory process. Certain substances, such as interleukin 1-β (IL-1β), released from the muscle injury act as intercellular messengers, start the process of inflammation and repair [4]. Moreover, tumor necrosis factor-alpha (TNF-α) is an important mediator of the inflammatory response after injury [5] whereas activation of PPAR, an anti-inflammatory protein, suppresses pro-inflammatory processes [6,7]. As a result of muscle injury, localized vasodilatation induced by two mechanisms comes about through the release of histamines from the cells present within the damaged area and by activating the route of the vascular endothelial growth factor and nitric oxide (VEGF-NO) [8]. VEGF is the most important capillary growth factor in skeletal muscle [7] and is essential to basal capillarization in the tissue and increased capillary growth in response to different mechanical stimuli [9].

Electrical stimulations are likely to serve as an integrator to organize cells into structured tissues in wound healing, development and tissues regeneration. Because cells possess signaling systems that make for electric stimulation, the exogenous application of therapeutic currents for wound healing is considered to have effects as well. The difficulties lie in the technical details such as types of electrodes, stimulation parameters, stimulation position, and the variability of intrinsic resistance [10].

The EPI® technique is an ultrasound guided physiotherapeutic and medical technique that consists in causing, by means of a galvanic current transmitted through an acupuncture needle, localized lysis in the damaged and/or degenerated tissue [11-13]. The application of a galvanic current brings about a chemical reaction, which causes the dissociation of molecules of sodium chloride and water. This process results in the formation of molecules of sodium hydroxide, which cause the destruction of the damaged tissue and activate the inflammatory repair response. The application of EPI® can stimulate the inflammatory response and promote wound healing in degenerated patellar tendon in rats [11] and has proven effective in the treatment of chronic patellar tendinopathy [12,13].

Currently there is no published basic research relative to the effect on muscle tissue injury upon applying this treatment. Accordingly, the objective of this study was to determine whether the application of EPI® therapy could have a beneficial effect on damaged muscle. An experimental design was carried out with the EPI® treatment after 7 days of Notexin-induced injury. Notexin has been described as inducing necrosis of skeletal muscle fibers in experimental inflammation models. Notexin, a presynaptic phospholipase A_2_ neurotoxin isolated from snake venom, produced inflammatory events associated with enzymatic activity and the release of arachidonic acid metabolites or mechanism related to phospholipid hydrolysis [14].

The experimental hypothesis is that the application of intratissue percutaneous electrolysis therapy after Notexina induced muscle damage causes muscular effects that may be conducive to the recovery of injured muscle tissue.

## Methods

Twenty-four Sprague-Dawley rats weighing 250-300g were divided into four groups. To induce muscle injury, 200 μl of Notexin was injected intramuscularly at a concentration of 10 μg/ml in the quadriceps of the left extremity, causing total degeneration of the muscle. As control, a group of rats (n = 6) were injected with 200 μl of saline solution. At seven days, rats were sacrificed and samples were obtained to determine the effects of Notexin-induced muscle injury. To study the effects of EPI® treatment on tissue injury, a specific approved EPI® device (EPI Advanced Medicine, Barcelona, Spain) was used. The following protocol was performed: on day seven of Notexin-induced muscle injury, one group of rats (n = 6) were treated with EPI®. This treatment consists in the application of a continuous current of 4 pulses at an intensity of 3 mA for 5 seconds conveyed to the muscle. As an electrode, an acupuncture needle with a diameter of 0.32 mm was used. To study how the injury evolves without receiving EPI® treatment, another group of rats (n = 6) was maintained dover 14 days after Notexin-induced injury. Previous to each treatment, rats were anesthetized intraperitoneally with sodium pentobarbital (90 mg/kg). The evolution of the muscle tissue injury was assessed by means of ultrasound images. The same evaluations were carried out after seven days of EPI® treatment. After the protocol, rats were sacrificed and muscle tissue was removed from the treatment area and samples were analyzed by using Western blot. Additionally blood samples were collected to detect TNF-α and IL-1β cytokines plasma levels with ELISA. The Ethical Committee of the University of Medicine of Valencia, Spain (A1301314899794) approved the study. All animal procedures were carried out in accordance with the European legislation on the use and care of laboratory animals (CEE 86/609).

**Figure 1:**
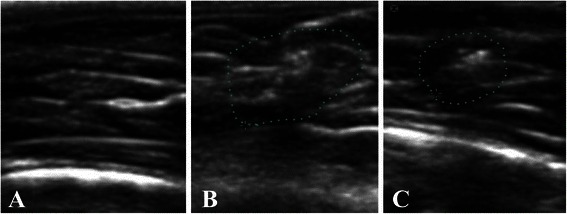
Comparison control tissue **(A)**, muscle tissue 21 days after injury induction with Notexina **(B)** and the effect of the application of EPI® from7 days of the induced lesion **(C)** in ultrasound imaging (US). It is possible to observe the area of disruption in the middle portion of the quadriceps muscle of rats from 21 days of the induced lesion (surrounded area), compared to normal tissue from the same area **(B)**. Image **(C)** shows an area of less disruption in the same muscle portion treated with EPI® from 21 days after induction of injury (surrounded area).

Ultrasonography was performed before and after EPI® treatment to follow up on the muscle tissue injury induced by Notexin (Figure 1). This examination was performed according to the protocol previously described [15].

Plasma levels of cytokines IL-1β and tumor necrosis factor-α (TNF-α) were determined with ELISA kits (Thermo Scientific Laboratories, Rockford, USA) following manufactorer’s recommendations.

Muscle tissues were homogenized in a lysis buffer of (in mM) 50 Tris-HCl, 125 NaCl, 1 EDTA, 1 EGTA and 1% Nonidet (NP-40 containing 5% Complete Mini-tab cocktail proteinase inhibitor (Roche Biochemicals). It was then centrifuged at 10000 rpm for 15 min at 4°C. The protein concentration was determined using a modified Lowry method. Protein was resolved in 12% SDS-PAGE and electrophoretically transferred onto a PVDF-membrane using a Mini Trans-Blot cell (BioRad laboratories, California). Membranes were put in blocks in 5% skim milk for 1 hour at room temperature and then incubated with the corresponding antibodies following the manufacturer’s recommendations. After washing, the membranes were incubated with horseradish peroxidase-conjugated secondary antibody (Sigma Aldrich). The blots were then visualized using a InmunostarTM HRP Substrate Kit (BioRad), again, in accordance with manufacturer’s instructions. The relative densities of the bands were analyzed using Image Gauge v4.0, Fujifilm. The proteins were normalized with tubulin. Monoclonal anti-vascular endothelial growth factor (VEGF) (1:500), anti-vascular endothelial growth factor receptor 1 (VEGF-R1) (1:500), anti-PPAR-γ (1:500) and anti-tubulin (1:1000) were used.

For statistical analysis, data are expressed as mean ± standard deviation (SD). An analysis of variance (ANOVA factor) was performed to analyze the relationships within and between variables. Post-Hoc and Dunnet tests were also done to compare the different groups with the control group and the Scheffe test was used to compare all groups. A probability value of less than 0.05 was considered significant.

## Results

**Figure 2:**
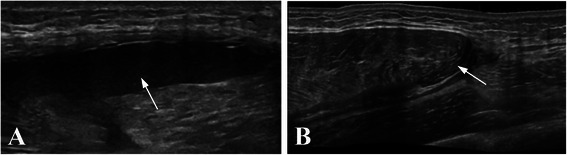
Longitudinal ultrasound images of left rat quadriceps. After 7 days treated with Notexin **(A)**, an anechoic image with fluid collection (arrow) indicating muscle lesion was observed. After EPI® treatment **(B)** a complete resorption of the haematoma with muscle repair (arrow) can be seen.

Notexin produced tissue injury characterized as an anechoic ultrasound image with fluid collection corresponding to a muscle lesion (Figure 2A). Treatment with EPI® produced resorption of the fluid and repair without scar tissue thickening (Figure 2B).

**Figure 3:**
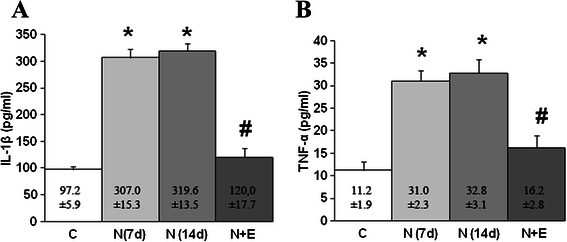
Plasma levels of IL-1β **(A)** and TNF-α **(B)** in control (C), Notexin (N7d, N14 d) and Notexin + EPI (N + E) groups. Values were measured by ELISA assay as indicated in methods. Data are mean ± SD of six independent experiments. *p < 0.05 *vs* control group; # *p* < 0.05 vs both Notexin groups.

The levels of TNF-α and IL-1β pro-inflammatory factors in Notexin injured rats showed a significant increase (p < 0.05) in plasma concentration relative to the control. In addition, a significant decrease in the concentration of TNF-α and IL-1β was observed when the Notexin + EPI group and the Notexin group (p < 0.05) were compared. So, the application of the EPI® treatment after Notexin provoked the decrease of both TNF-α and IL-1β to control levels (Figure 3A and B). After 14 days of Notexin treatment without EPI® application, the values of cytokines continued increased (Figure 3A and B). These results rule out spontaneous recovery of the muscle damage.

**Figure 4:**
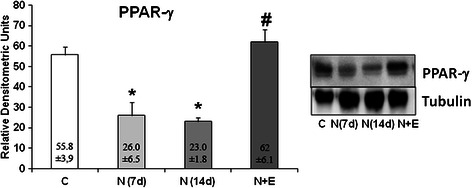
PPAR-γ protein expression (relative densitometric units) in control (C), Notexin (N7d, N14 d) and Notexin + EPI (N + E) groups. Values were determined in left rat quadriceps muscles by Western blot. A representative inmunoblot is shown and tubulin was used as control amount of protein. Data are mean ± SD of six independent experiments. *p < 0.05 *vs* control group; # *p* < 0.05 vs both Notexin groups.

Similarly, Notexin-induced injury decreases PPAR-γ expression values (p < 0.05) in rat quadriceps muscle. The application of EPI® increased PPAR-γ expression and were returned to the values of the control, showing that EPI® treatment produces an improvement in anti-inflammatory PPAR-γ protein (Figure 4). Furthermore, at 14 days of Notexin treatment without EPI® application, PPAR-γ protein expression remains decreased, thus indicating that an increase in PPAR-γ protein expression is not spontaneous but due to the EPI® treatment.

**Figure 5:**
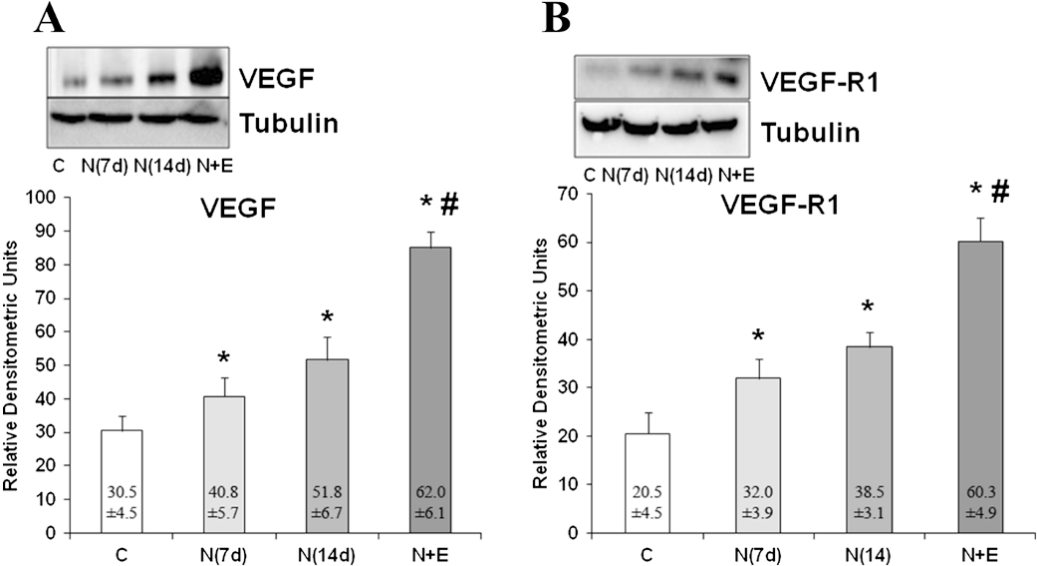
Analysis of VEGF and VEGF-R1 proteins. VEGF **(A)** and VEGF-R1 **(B)** protein expression in control (C), Notexin (N7d, N14 d) and Notexin + EPI (N + E) groups were determined by Western blot. Values were determined in left rat quadriceps muscles. In each panel, a representative inmunoblot is shown and tubulin was used as control amount of protein. Data are mean ± SD of six independent experiments. *p < 0.05 *vs* control group; # *p* < 0.05 vs both Notexin groups.

Notexin (7 and 14 days) treatment produced an increase in both VEGF and VEGF-R1 protein expression compared with the control (p < 0.05). Furthermore, EPI® treatment significantly potentiated the increase in VEGF and VEGF-R1 protein expression induced by Notexin (Figure 5).

No adverse events were presented during the study.

## Discussion

The main findings of this study is that EPI® applied after Notexin-induced muscle injury in rats decreases the production of the inflammatory mediators TNF-α and IL-1β, increases the protein expression of anti-inflammatory factor PPAR-γ and the angiogenic involved proteins VEGF and VEGF-R1.

An increase in the TNF-α plasma levels was described in the first days of tissular injury [16,17] and remained elevated due to its action on cellular necrosis [18]. TNF-α disrupts the differentiation process and can promote cell catabolism thereby accelerating protein degradation [5]. Furthermore, TNF-α inhibits myogenesis through redox-dependent and independent pathways [19]. One potential mechanism by which TNF-α might directly stimulate catabolism is by inhibiting myoblast differentiation, an action that might limit the regenerative response of satellite cells to muscle injury [5]. A second mechanism, apoptosis, appears less important. The third mechanism consists in a direct catabolic effect on muscle tissue. In a muscular cell culture, TNF-α directly decreases total muscle protein and the loss of muscle-specific proteins, including adult fast-type myosin heavy chain [5,19].

Our data shows an increase in the plasma level of TNF-α due to Notexin-induced injury. EPI® treatment normalized the levels of TNF-α to reach control group values. By contrast, in the group of rats without EPI® treatment, the TNF-α levels remained elevated with respect to the control group at 14 days after application.

TNF-α action is also sensitive to other ligand/receptor interactions (e.g. interleukin-1 and interleukin-6). Notexin caused a significant increase of IL-1β compared to the control group. The maintenance of IL-1β over time has been associated with its condition as a pro-inflammatory cytokine more than for its action on tissue necrosis [16]. Furthermore, IL-1β induces inhibition of protein synthesis in skeletal muscle [20]. EPI® treatment returns IL-1β plasma levels to normal values. On the contrary, after 14 days of application without EPI®, the levels of IL-1β remain significantly high compared to control values. Taken together, the results indicate that EPI® treatment is effective in diminishing pro-inflammatory mediators. Further studies are needs to determine the mechanisms involved in the inflammatory effects of EPI® treatment. Besides that, EPI® decreases pro-inflammatory mediators and anti-inflammatory proteins may also be activated. PPAR-γ has been recognized as playing a fundamental role in the immune response through its ability to decrease the expression of pro-inflammatory genes [21]. It also increases the expression levels of genes that are involved in anti-inflammatory effects and tissue repair [22,23]. Furthermore, PPAR-γ induces the expression of VEGF and its receptors in cultured cardiac myofibroblasts [24]. Our data indicated that Notexin produced a significant decrease in PPAR-γ protein expression, similar at 7 and 14 days, compared with control. EPI® treatment significantly increases PPAR-γ protein expression reduced by Notexin and returns levels to control values. In addition, PPAR-γ promotes the myocellular storage of energy by increasing fatty acid uptake and esterification while simultaneously enhancing insulin signaling and glycogen formation, which have beneficial effects on metabolic health and therefore on tissue repair [25].

Electrical stimulation has multiple effects in directing cell division, vascular endothelial cells, angiogenesis and endothelial migration, all of which are important elements in wound healing [10]. Vascular endothelial growth factor (VEGF) is a paracrine factor. Its main function is to promote angiogenesis by improving cellular survival, inducing proliferation and enhancing the migration and invasion of endothelial cells. Skeletal muscle fibers can control capillary growth by releasing VEGF from intracellular vesicles during contraction [26]. Recent evidence suggests that VEGF has effects on skeletal muscle regeneration by stimulating the myogenic differentiation of muscle-derived stem cells [27,28].

Our results indicate a clear induction of VEGF protein expression after Notexin-induced damage. These results are in accordance with a greater production of VEGF in damaged tissue than in normal tissue [29]. Furthermore, VEGF-R1, the more actively induced receptor by tissue injury, is also increased as has been described in trauma patients [30]. EPI® treatment further significantly increases both VEGF and VEGF-R1 thus suggesting an active role in maintaining blood flow in the microcirculation and also may increase the systemic level of soluble anti-inflammatory and cytoprotective mediator events that can improve the recovery from injury [30].

Despite the many treatments proposed to treat muscle injuries, the rate of re-injury is still very high. This is probably due to the fact that a greater understanding and analysis of the type, size and location of the lesion in each case [31] is required.

Some authors argue that the size of the lesion correlates with the time the patient will need to return to competition [32]. By contrast, other study groups suggest that neither the presence of ultrasound findings nor the size of them correlate with the time needed to return to competition. Thus, the prognosis for muscular injuries should not be guided by these results alone [33,34].

Although the number of cases may be considered low, the difference between the variables studied was very high. Therefore, sufficient power was obtained so as to detect differences with a significance ranging from 55 to 58% for VEGF and VEGF-R1 variables as well as from 88% to 100% in TNF and IL-1B variables.

The work has some limitations such as the use of rats. As such, it might not be possible to extrapolate the result to humans. In spite of that possibility, rats have been used in many valid experimental studies [14-17,20,27]. Another limitation is the lack of a histological or functional evaluation, which could give physiological relevance to the interpretation of the data presented [35]. The electrolysis and/or sodium hydroxide produced by the EPI® technique may interfere with IL-1beta and TNF-alpha values, affecting the existing cytokines. Therefore, we wait 7 days after the EPI® technique application to see its beneficial effects. Cytokines from the cells at the local position will be produced chronically and maintained over time when inflammation and damage is present. We detect a reduction of pro-inflammatory cytokines after EPI® induction. Thus, cells are probably in a state of less inflammation with less cytokine production in comparison to cells without the EPI® technique.

Despite the limitations exposed, the present work is the first investigation on the effect of EPI® on muscle tissue that shows the biomolecular mechanisms triggered by the application of the same. This experimental work is the basis upon which clinical trials to confirm the effectiveness of the EPI® in humans should be developed.

## Conclusion

The application of EPI® on rat muscle previously injured with Notexin causes a significant decrease in pro-inflammatory mediators like TNF-α as well as IL-1β levels. On the other hand, the application of EPI® produced an increase in the expression of anti-inflammatory proteins (PPAR-γ) and also increases VEGF and VEGF-R1 expression. Therefore, the use of EPI® may affect inflammatory mediators in damaged muscle tissue and influence the new vascularization of the injured area. These results suggest that EPI® might represent a useful new therapy for the treatment of muscle injuries.

Although our study in rats may represent a valid approach to evaluate EPI® treatment, studies designed to determine how EPI® treatment may affect recovery of injury in humans are needed.
